# Tree identity and diversity directly affect soil moisture and temperature but not soil carbon ten years after planting

**DOI:** 10.1002/ece3.8509

**Published:** 2022-01-12

**Authors:** Marc‐Olivier Martin‐Guay, Michaël Belluau, Benoit Côté, Ira Tanya Handa, Mark D. Jewell, Rim Khlifa, Alison D. Munson, Maxime Rivest, Joann K. Whalen, David Rivest

**Affiliations:** ^1^ Institut des Sciences de la Forêt Tempérée (ISFORT) Université du Québec en Outaouais (UQO) Ripon Quebec Canada; ^2^ Département des Sciences Biologiques Université du Québec à Montréal (UQÀM) Montréal Quebec Canada; ^3^ Department of Natural Resource Sciences (NRS) McGill University Montréal Quebec Canada; ^4^ Department of Biology McGill University Montréal Quebec Canada; ^5^ Département Science et Technologie Université TÉLUQ Montréal Quebec Canada; ^6^ Département des Sciences du Bois et de la Forêt Université Laval Québec Quebec Canada

**Keywords:** biodiversity and ecosystem services, IDENT, soil carbon sequestration, tree diversity experiment, tree effects on soils

## Abstract

1. Soil C is the largest C pool in forest ecosystems that contributes to C sequestration and mitigates climate change. Tree diversity enhances forest productivity, so diversifying the tree species composition, notably in managed forests, could increase the quantity of organic matter being transferred to soils and alter other soil properties relevant to the C cycle.
2. A ten‐year‐old tree diversity experiment was used to study the effects of tree identity and diversity (functional and taxonomic) on soils. Surface (0–10 cm) mineral soil was repeatedly measured for soil C concentration, C:N ratio, pH, moisture, and temperature in twenty‐four tree species mixtures and twelve corresponding monocultures (replicated in four blocks).
3. Soil pH, moisture, and temperature responded to tree diversity and identity. Greater productivity in above‐ and below‐ground tree components did not increase soil C concentration. Soil pH increased and soil moisture decreased with functional diversity, more specifically, when species had different growth strategies and shade tolerances. Functional identity affected soil moisture and temperature, such that tree communities with more slow‐growing and shade‐tolerant species had greater soil moisture and temperature. Higher temperature was measured in communities with broadleaf‐deciduous species compared to communities with coniferous‐evergreen species.
4. We conclude that long‐term soil C cycling in forest plantations will likely respond to changes in soil pH, moisture, and temperature that is mediated by tree species composition, since tree species affect these soil properties through their litter quality, water uptake, and physical control of soil microclimates.

## Graphical abstract

Soil abiotic properties were affected by tree diversity and identity. Tree composition had indirect effects on soil carbon through soil microclimate.

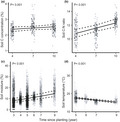

## 1 INTRODUCTION

Globally, forests are a net carbon (C) sink that has accumulated 2.0–2.8 Pg y^−1^ between 1990 and 2007, especially in temperate and boreal regions (Pan et al., 2011). Given that the soil organic carbon (SOC) pool is larger and has a longer residence time than that of the living plant biomass (Scharlemann et al., 2014), the transfer of C from trees into the SOC pool is crucial. This pool receives C inputs in the form of litterfall, below‐ground litter, and rhizo‐deposition, and it loses C through soil faunal and microbial respiration, leaching of dissolved organic matter, and erosion (Mayer et al., 2020). Each tree species supplies a particular quantity of inputs with unique chemical compositions and also affects soil biophysical properties and soil microbial communities (Vesterdal et al., 2013); thus, species composition and interspecific interactions will influence the dynamics of this pool.

Tree mixtures usually have greater SOC concentrations and larger SOC stocks than their corresponding monocultures (Chen et al., 2020), thereby suggesting a greater C input: C output ratio. There is growing evidence that tree diversity positively affects above‐ground productivity (Liang et al., 2016; Paquette & Messier, 2011; Vilà et al., 2013; Zhang et al., 2012). This diversity effect will increase litterfall, but litter decomposition and soil respiration rates can be higher in diverse communities (Handa et al., 2014; Jewell et al., 2017). Increased soil respiration can be explained by the fact that plant mixtures usually show increased microbial biomass and respiration (Chen et al., 2019). However, soil microorganisms have conflicting roles regarding the fate of plant‐derived soil organic matter (SOM). While they contribute to SOC losses through respiration, their necromass contains recalcitrant compounds that will form stable SOC (Buckeridge et al., 2020; Cotrufo et al., 2013; Kleber et al., 2011; Ma et al., 2018; Schmidt et al., 2011). For example, the Jena Experiment (Germany), which manipulated plant diversity, showed that SOC storage increased with species richness due to an increased microbial anabolism leading to an efficient transfer of SOM from fast‐cycling SOC pools (labile plant residues) to slow‐cycling SOC pools (recalcitrant microbial necromass) (Lange et al., 2015). Although diversity positively affects SOC, Mayer et al. (2020) assert that tree identity, both taxonomic (i.e., species or higher‐rank taxa such as angiosperms and gymnosperms) and functional (i.e., classifying trees based upon functional traits), is more important than diversity when explaining differences in SOC stocks. Moreover, identity and diversity effects can be entangled since diversity increases the chance of including keystone species, which have a strong effect on productivity or other ecosystem functions (Huston, 1997; Loreau, 1998; Loreau & Hector, 2001). Therefore, diversity and identity effects occur at the same time and both should be considered when investigating tree effects on soils.

Functional traits can explain why most coniferous‐evergreen species stands accumulate SOC in the organic layer, most likely due to their recalcitrant above‐ground litter, in contrast to the SOC accumulation in mineral soil under broadleaf‐deciduous species, which could be explained by increased bioturbation by a more abundant soil macrofauna (Mayer et al., 2020; Vesterdal et al., 2013). Litter chemistry affects other soil properties, given that soil pH and the concentration of base cations is lower in soils that had inputs of coniferous litter (Millar, 1974). For example, a common‐garden experiment using temperate species monocultures has shown that the litter concentration of calcium can positively influence pH and the abundance of earthworms, while reducing SOC in the organic layer (Reich et al., 2005). Lower pH conditions may be suboptimal for extracellular enzymes (Sinsabaugh et al., 2008) and limit the action of soil microorganisms and fauna, which will affect litter decomposition rates and SOC accumulation. Given that experiments that add N to the soils of north temperate forests have shown increased SOC (Nave et al., 2009), tree species inputs with reduced C:N ratios could favor SOC accumulation, perhaps due to increased microbial growth and an accumulation of recalcitrant compounds originating from microbial necromass. Therefore, tree functional identity likely affects soil chemistry, but species interactions could also be relevant. The positive effect of diversity on productivity (Liang et al., 2016) could increase the supply of base cations and result in higher soil pH. Below‐ground complementarity in resource acquisition, which is a recurring hypothesis when explaining this positive effect (Mueller et al., 2013; Oram et al., 2018), could mean litter that is richer in N.

Functional traits also will determine the extent to which trees create microclimates by shading the soil surface and through water uptake. Some trees form a thick litter layer that acts as a physical barrier to moisture evaporation and infiltration, as well as insulation that limits the effects of air temperature fluctuations (Facelli & Pickett, 1991). An increase in soil temperature will stimulate decomposition, based upon greater soil respiration in soil‐warming experiments (Sun et al., 2011). For example, surface temperatures at night and wood decomposition were shown to be influenced by tree identity in a diversity experiment with trees, while tree species richness had no effect (Gottschall et al., 2019). Conversely, lower soil moisture is expected to limit microbial activity in forest soils, based upon the decline in microbial biomass that has been measured in forest precipitation–interception experiments (Zhou et al., 2018).

**FIGURE 1:**
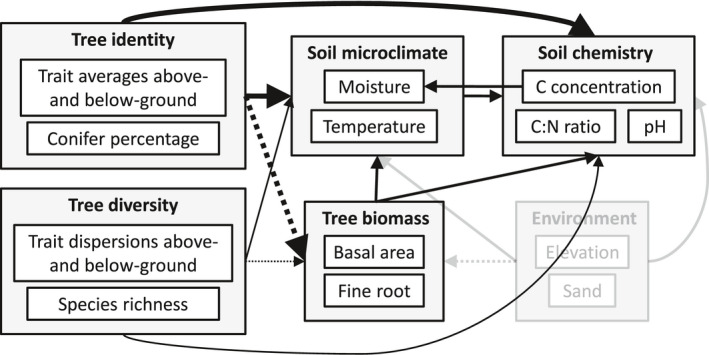
Conceptual diagram showing the different variables that were measured in our study and the relationships that were tested. Diversity and identity effects on biomass are shown (dotted arrows) but they have been tested in previous studies (Archambault et al., 2019; Tobner et al., 2016). Stronger identity effects compared to diversity effects are hypothesized and shown by arrow sizes. Environmental control variables are shaded

Using a ten‐year‐old common‐garden experiment manipulating tree diversity (IDENT—Montreal, QC), we tested how tree diversity and identity at different times following tree planting can affect soil C concentrations and associated soil properties, viz., C:N ratio, pH, moisture, and temperature. Even though tree identity and diversity can directly affect soil chemistry (C, C:N ratio, and pH), we explored the possibility of indirect effects through alterations to soil microclimate (moisture and temperature), which can rapidly be affected by trees (Figure 1). We also tested the direct effects of tree biomass (above‐ and below‐ground) to identify the mechanism by which trees influence soil properties.

Many studies that are relevant to the present research were conducted on this site and helped us to choose directions for the hypotheses. There was a gradient of above‐ground productivity in the plots, leading to greater inputs in some mixtures compared to their corresponding monocultures (Tobner et al., 2016). Yet, litter decomposition rates were higher under tree mixtures and when litter was diverse in terms of N concentrations (Jewell et al., 2017). Below‐ground inputs also varied among mixtures and monocultures due to the dominance of species with less root biomass in mixtures (Archambault et al., 2019; Martin‐Guay et al., 2020). Tree diversity and composition exerted only a weak effect on the compositions of soil bacteria and fungi (Rivest et al., 2019), but microbial communities in mixtures had greater biomass and basal respiration, while utilizing a greater number of carbon sources (Khlifa et al., 2017). Consistent with increased microbial activity, tree diversity also had a positive effect on surface CO_2_ emissions (Jewell et al., 2017). Hence, our first hypotheses were:

1. Tree diversity (species richness or functional diversity) is positively related to soil C concentration because greater diversity increases tree productivity and C inputs to the soil.
2. Tree diversity creates soil properties that are favorable to microbial activity, that is, a reduced C:N ratio, near‐neutral pH, higher soil moisture, and higher temperature.

When comparing microbial activity among monocultures, greater activity was observed under deciduous species (Khlifa et al., 2017). Tree identity was also the main driver of productivity above‐ and below‐ground, with deciduous species being the most productive ones in monocultures and the most dominant ones in mixtures (Archambault et al., 2019; Tobner et al., 2016). Hence, our final hypotheses were:

3. Tree identity (functional or taxonomical) influences soil properties because a greater abundance of less productive conifers will not contribute to SOC accumulation and will inhibit microbial activity, that is, greater C:N ratio, acidic pH, lower soil moisture, and lower temperature.
4. Identity effects are stronger than diversity effects for the five soil variables due to keystone species and their specific traits strongly affecting ecosystem functioning.
5. Tree diversity and identity effects strengthen with time due to cumulating effects of trees on soils and increasing interspecific interactions.

## 2 MATERIALS AND METHODS

### 2.1 Experimental design

The experimental site is located on the Macdonald Campus Farm of McGill University (Sainte‐Anne‐de‐Bellevue, QC, Canada, 45°28′N, 73°45′W, 36 m). The soil is a Humic Gleysol (Typic Endoaquent, USDA 7th approx.) with a pH of 6.3 in the 0‐ to 20‐cm sandy loam layer (average 78% sand, 6% silt, and 16% clay; Rivest et al., 2015). The experiment is part of IDENT (International Diversity Experiment Network with Trees; Tobner et al., 2014), which is linked to the larger network TreeDivNet (Paquette et al., 2018; Verheyen et al., 2016). The site was established in spring 2009, when nearly 15,000 trees were planted on less than 1 ha that was formerly under agricultural production (i.e., different cash crops in rotation). The trees were distributed in Cartesian grid‐plots containing 64 individuals (8 × 8 rows; 50‐cm spacing) and separated by corridors (1.25 m) to reduce interaction between tree plots. These corridors were trenched to a depth of 30 cm during the summers of 2011 and 2012 to prevent interaction between the roots of neighboring tree communities. The area is relatively flat and precise elevation (micro‐topography) was measured at the plot level using standard surveying equipment (cm; total station theodolite) to account for minor depressions and bumps (maximal difference of 36 cm between the centers of two plots), since micro‐topography affects water availability for growth (Tobner et al., 2016) and biomass allocation (Martin‐Guay et al., 2020).

Treatments in this study were twelve monocultures, fourteen two‐species mixtures, and ten four‐species mixtures of randomly distributed species (with restrictions to prevent clumping). These species are found in North American temperate forests and included five broadleaf species (*Acer rubrum* L., *A. saccharum* Marshall, *Betula alleghaniensis* Britton, *B. papyrifera* Marshall, and *Quercus rubra* L.) and seven coniferous species (*Abies balsamea* [L.] Miller, *Larix laricina* [DuRoi] K. Koch, *Picea glauca* [Moench] Voss, *P. rubens* Sargent, *Pinus resinosa* Aiton, *P. strobus* L. and *Thuja occidentalis* L.). Each treatment was replicated in four randomized blocks. For each level of species richness (SR), mixture compositions were randomly selected from all possible combinations of species and placed along a functional diversity (FD) gradient. FD was calculated as a multidimensional functional dispersion index (Laliberté & Legendre, 2010) using twelve functional traits. From this, eight levels of FD were determined that were used to classify mixtures (for more detailed information regarding the design, see Tobner et al., 2014, 2016).

### 2.2 Soil measurements

Three independent measurements of total C and total N concentrations and pH were made on the mineral soil layer from each plot. Total C includes both soil organic carbon (SOC) and inorganic carbon, thus "soil C" and not "SOC" was used when addressing our results. Soil samples were composites of five cores per mixture plot and three cores per monoculture plot in October 2012 (diameter = 7 cm, depth = 0–15 cm; see Khlifa, 2016); 20 cores per plot in September 2015 (diameter = 2 cm, depth = 0–10 cm; see Rivest et al., 2019); and five cores per plot in May 2019 (diameter = 2 cm, depth = 0–10 cm). Samples were air‐dried and sieved (2 mm) to remove roots and debris. Total C and N concentrations were measured by high‐temperature dry combustion with a TruMac CNS analyser (LECO Corp., Saint Joseph, MI, USA). Given that bulk density data were unavailable, we could not calculate SOC stocks which more accurately represent carbon sequestration compared to soil C concentration. However, the experiment was a microcosm without immediate application to actual forest ecosystems (plantations of these species at this density do not exist), and, therefore, accurate estimates of SOC stocks are not relevant. Rather, the main focus is testing hypotheses linking trees to the C cycle, for which soil C concentrations are sufficient. Soil pH was measured in slurries with a 1:2 soil‐to‐liquid ratio (0.01 M CaCl_2_ for 2012 and deionised water for 2015 and 2019; values for these latter two years were converted to pH‐CaCl_2_ using Ahern et al.'s (1995) fourth equation, which is suitable for the pH conditions in this study according to Henderson and Bui (2002)). Soil texture in each plot was characterized with the hydrometer method (Bouyoucos, 1962) in October 2012.

Soil moisture was surveyed eleven times, that is, three times in 2011 (June 27, July 21, and August 16), once in 2012 (July 20), four times in 2013 (July 16–17, July 26, August 7, and August 23), once in 2014 (May 22), once in 2015 (July 17), and once in 2017 (July 3–4). Moisture was assessed with a FieldScout TDR 300 (Spectrum Technologies, Aurora, IL, USA) with 12‐cm rods at 5 to 9 different locations in each plot, depending upon the survey. Soil temperature was measured using one permanently placed (10‐cm depth) thermocouple at the center of each plot (Barnant Co., Barrington, IL) on the same date as soil moisture between 2013 and 2017. Another soil temperature survey was performed on three days in 2015 (June 25–26 and July 2). Data from each survey were standardized to account for antecedent weather conditions based upon the total precipitation in the preceding 10 days and the average air temperature in the past 5 days (Appendix S1).

### 2.3 Basal area and fine root biomass

In each autumn from 2009 to 2018, ground‐level (5 cm) diameter was measured for each of the 64 trees (still alive) per plot using digital calipers and ground‐level basal area was estimated for each species within each plot. We assumed that the effects of trees on soil C concentration, C:N ratio and pH would be cumulative, that is, due to the effect of accumulated litterfall and its decay over the years. Therefore, cumulative ground‐level basal area was calculated for each species within each plot for fixed time intervals (shown in Figure S3).

In October 2012, standing fine root (<2 mm) biomass was evaluated in each monoculture plot in the same cores that were used for C, N, and pH (Archambault et al., 2019). Although fine roots were sampled to 40‐cm depth, only roots from the top 10‐cm layer were used here. At the same time, a modified ingrowth core method (Lund et al., 1970) characterized annual fine root production (see Archambault et al., 2019). In June 2012, two soil cores (diameter = 8 cm, depth = 0–15 cm) per plot were extracted and refilled with root‐free soil. In June 2013, the ingrowth cores were removed and sieved to recover fine roots. For both samplings, that is, standing biomass and annual production, all recovered roots (dead or alive) were then washed, oven‐dried at 60 or 65 °C, and weighed. Average standing biomass and annual production per core were scaled up to the entire plot area (3.5 m × 3.5 m). A ratio between each fine root metric and basal area was calculated for each monoculture treatment. These ratios permitted the estimation of standing fine root biomass and annual production from 2009 to 2018. These estimates were cumulated when linked to soil C concentration, C:N ratio, and pH (as shown in Figure S3).

### 2.4 Functional trait metrics

**FIGURE 2:**
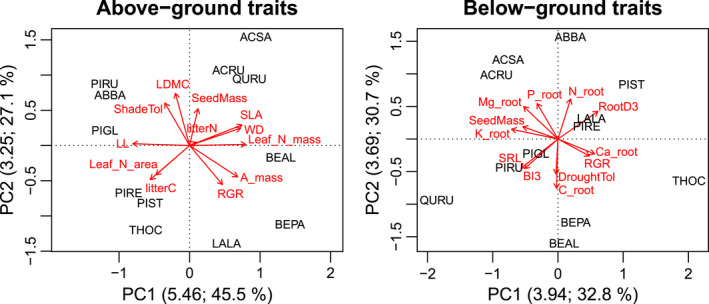
Principal component analyses (PCA) for above‐ground and below‐ground functional traits. Scaling permits to represent accurately the correlations between traits. Values in parentheses are the eigenvalues followed by the proportions of variance explained for each axis. Traits included in both PCAs are: annual relative growth rate (RGR; year^−1^) and seed mass (g 1000^−1^seeds). Included only in the above‐ground PCA, there are six leaf traits: net maximum photosynthesis per mass (A_mass; µmol g^−1^ s^−1^), dry matter content (LDMC; mg g^−1^), N content per mass (Leaf_N_mass; mg g^−1^) or area (Leaf_N_area; g m^−2^), longevity (LL; month), specific area (SLA; mm^2^ mg^−1^); two litter traits: C and N concentrations (litterC and litterN; mg g^−1^); shade tolerance (ShadeTol; from 0‐intolerant to 5‐tolerant); and wood density (WD; g cm^−3^). Included only in the below‐ground PCA, there are three fine root morphological traits: branching intensity (from 1^st^‐ to 3^rd^‐order; BI3; no. tips g^−1^), average diameter (from 1^st^‐ to 3^rd^‐ order; RootD3; mm), and specific length (SRL; m g^−1^); six fine root chemistry traits: C, N, P, K, Ca and Mg concentrations(Root_[element abbreviation]; mg g^−1^); and drought tolerance (DroughtTol; 0‐ intolerant to 5‐tolerant). The species codes include the first two letters of the genus, followed by the first two letters of the species. Data with different units were standardized prior to PCA

Many functional traits are strongly correlated, reflecting life‐strategy trade‐offs, for example, the “fast‐slow” plant economic spectrum (Reich, 2014). To avoid multicollinearity problems and spurious relationships, each trait cannot be tested separately for its effects on soil variables; thus principal component analysis (PCA) was done separately for above‐ground functional traits and below‐ground traits (Figure 2), reducing the traits to a few dimensions. Traits were transformed to achieve normality and standardized prior to PCA. Both PCAs included relative growth rate and seed mass since they are life‐strategy traits related to traits above‐ and below‐ground. The above‐ground PCA included six leaf traits, viz., net maximum photosynthesis per mass, dry matter content, N content per mass or area, longevity and specific area; two litter traits, that is, C and N concentrations; shade tolerance; and wood density. The below‐ground PCA included three fine root morphological traits, viz., branching intensity (from 1st‐ to 3rd‐order), average diameter (from 1st‐ to 3rd‐order), and specific length; six fine root chemistry traits, viz., C, N, P, K, Ca, and Mg concentrations; and drought tolerance. Data for these traits were derived from an online database that was dedicated to the IDENT project (https://doi.org/10.6084/m9.figshare.13118132.v1), except for fine root chemistry traits (Khlifa et al., 2017) and annual relative growth rate. This rate was calculated using cylindrical volumes (*basal diameter* × *height*) of individual trees in monocultures (*ln*(*2014 volume*/*2009 volume*) / *5 year*). All of these traits were measured in situ in the first five years after planting, except for seed mass, leaf photosynthesis, leaf N, leaf longevity, wood density, shade, and drought tolerances. Shade and drought tolerances were the only traits that were based upon multifactor rankings and not direct measurements (Niinemets & Valladares, 2006).

In both PCAs, the eigenvalues of the first two axes explained more variation than did random expectation (broken‐stick method). Species scores on these axes were used to calculate community‐weighted means (CWM) in every plot as a characteristic of their functional identity. Functional diversity relied upon these axis scores to calculate functional dispersions (FDis; Laliberté & Legendre, 2010). Each species weight in CWM and FDis calculations was based upon ground‐level basal area for above‐ground traits and estimated standing fine root biomass for below‐ground traits. Standing biomass was chosen instead of annual production since the former was measured with five cores in mixtures while the latter only relied upon two cores, which is limited when attempting to capture variability of each mixture. Since the N concentration in litter was uncorrelated (orthogonal) with axes 1 and 2 in the above‐ground PCA (*r* = .11 and .06, respectively) and its importance had been shown for litter decomposition and soil respiration (Jewell et al., 2017), we calculated CWM and FDis for this trait alone.

The first axis of the above‐ground trait PCA (Above1) clearly separates coniferous‐evergreen species from broadleaf‐deciduous species based mainly upon leaf traits. Note that *Larix laricina* is positioned in the axis midpoint as a deciduous conifer. The clear delineation between species groups explains why the variable conifer percentage is not present in the analyses that are presented here. Indeed, conifer percentage is strongly correlated with Above 1 (*r* > .85), which incurred problems of multicollinearity and prevented their simultaneous use. The functional trait approach was deemed superior to conifer percentage, which is merely a taxonomic variable at the phylum level. The second axis of this PCA (Above2) is more closely associated, not only with life‐strategy traits such as growth, shade tolerance, and seed mass, but also leaf dry‐matter content (Figure 2). The first axis of the below‐ground PCA (Below1) is mostly correlated with root K and Ca concentrations, root diameter, and specific root length. The second axis (Below2) is mostly correlated with root C, N, and P concentrations.

### 2.5 Light measurements

Understory light availability was determined in three summers when trees had fully flushed (9–25 July 2013; 19–25 August 2014; 28 July to 7 August 2015) using BF Sunshine sensors (Delta‐T Devices, Cambridge, UK). These sensors evaluated the amount of diffuse photosynthetically active radiation (PAR; µmol m^−2^ s^−1^) using an internal shading system limiting the effect of direct light on the device and, consequently, the effect of changing weather conditions. In each plot, ground‐level PAR was measured at five different locations, while simultaneous measurements were made in full sun just outside the experiment. With these two sets of data, fractions of PAR (fPAR) reaching the ground were calculated as:

$$\text{fPAR}=\frac{\text{ground}‐\text{level}\;\text{PAR}}{\text{above}‐\text{canopy}\;\text{PAR}}.$$

### 2.6 Statistical analyses

Tree functional diversity and identity were related to five different soil variables, that is, C concentration, C:N ratio, pH, moisture, and temperature, with general linear mixed‐effect models (see Table 1 where all tested relationships are shown). When explaining C concentration (*N* = 424), C:N ratio (*N* = 424), and pH (*N* = 427), the models took the following form:

(1) $$\text{Soil}\;\text{variable}\;∼\;\left(\begin{array}{c} \text{FDis}\;\text{Above}1+\text{FDis}\;\text{Above}2+\text{FDis}\;\text{Below}1+\text{FDis}\;\text{Below}2+\\ \text{FDis}\;\text{litterN}+\text{SR}+\text{Cumulated}\;G+\text{Cumulated}\;\text{RB}+\text{Cumulated}\;\text{RP}+\text{CWM}\;\text{Above}1+\\ \text{CWM}\;\text{Above}2+\text{CWM}\;\text{Below}1+\text{CWM}\;\text{Below}2+\text{CWM}\;\text{litterN}+\%\text{Moisture}+T^{\circ }+\\ \text{Elevation}+\%\text{Sand} \end{array}\right)\times \text{Time}+\text{random}\left(\text{Block}\right)+\text{random}\;\left(\text{Treatment}\right)$$

where Soil variable is soil C concentration, C:N ratio, or pH; SR is species richness; ground‐level basal area (G), root biomass (RB), and production (RP) are cumulated over the years (Figure S3). FDis and CWM are based upon species scores on the above‐ground PCA axes (Above1 and Above 2), species scores on the below‐ground PCA axes (Below1 and Below2), and species values for litter N concentration. %Moisture and T° are, respectively, average soil moisture and temperature over all years for each plot. Elevation and %Sand are control variables for micro‐topography at the plot level and soil texture, respectively. All single terms in parentheses were tested for an interaction with Time; Time is the number of years that trees have been growing together (i.e., 4, 7, or 10 y depending upon the sampling year), while the random effect structure takes into account the spatial correlation due to blocks and the correlation between plots having the same treatment (i.e., tree composition). Likelihood ratio tests were used to evaluate which predictor was significant. These tests compared the full model without interaction with a model having a deleted single term or with a model having an added interaction.

**TABLE 1:** Standardized regression coefficients (β) from the final general linear mixed‐effect models explaining soil C concentration, C:N ratio, pH, moisture, and temperature

Predictor	[C]	C:N ratio	pH	Moisture	Temperature
	*R^2^* = .05 *N* = 424	*R^2^* = .25 *N* = 424	*R^2^* = .11 *N* = 427	*R^2^* = .24 *N* = 9134	*R^2^* = .27 *N* = 1147
Tree diversity					
FDis Above1			.020**↗**	.052**↘**	
FDis Above2			.**064↗**	**−.189**	
FDis Below1			−.064**↘**		n/a
FDis Below2			−.002**↗**		n/a
FDis litterN			−.004**↗**	n/a	n/a
Species richness			.012**↗**		
Tree biomass					
Basal area^a^				−.043**↘**	
Root biomass^a^				.**062**	n/a
Root production^a^				**−.065↗**	n/a
Tree identity					
CWM Above1				.083**↗**	.**024**
CWM Above2				.**337↗**	.**010**
CWM Below1				.250**↗**	n/a
CWM Below2				.021**↘**	n/a
CWM litterN				n/a	n/a
Soil conditions					
%Moisture	n/a^b^			n/a	n/a
Temperature		−.046**↘**		n/a	n/a
fPAR	n/a	n/a	n/a	n/a	
Elevation			.**083**	**−.070↘**	
%Sand	**−.050**			**−.135↘**	
Time	.**065**	.**619**	−.006	.**264**	**−.095**

Note

These final models used only the significant variables, but all tested variables are shown (n/a indicates that the predictor was not tested in a specific model; see Equations (1), (2), (3)). Marginal *R* ^2^‐values are shown, each of which includes only the variance explained by fixed effects in each final model. Significance was evaluated using likelihood‐ratio tests and is shown with boldface coefficients or an arrow for significant interactions with time (α = .05 and false discovery rate method).

Abbreviations: Above1 and 2, first and second axis of the above‐ground trait PCA (see Figure 2); Below1 and 2, idem for the below‐ground trait PCA; CWM, community‐weighted mean; FDis, functional dispersion; fPAR, fraction of photosynthetically active radiation reaching the ground; litterN, litter N concentration.

^a^ Cumulated over the years in the models explaining soil C concentration, C:N ratio, and pH (see Figure S3).

^b^ Tested in a correlation.

In the model explaining soil C, it was inappropriate to test soil moisture since the direction of causality for these two variables is unclear (see Figure 1). Soil C concentration is intrinsically linked to soil organic matter (SOM) and, subsequently, to water‐holding capacity (WHC). Conversely, moisture could cause enhanced microbial activity and, consequently, enhanced C stabilization (soil microclimate affecting soil chemistry in Figure 1). Therefore, a Pearson's correlation test was performed using each plot average for soil moisture and soil C concentration across all samples (*N* = 144). This yields information regarding the link between these two variables, with no assumption about a particular causal relationship.

Testing functional identity and diversity effects on soil moisture (*N* = 9134) and temperature (*N* = 1147) followed the same procedure, with a few differences. First, G, RB, and RP were not cumulated over the years because it was assumed that tree density exerted more immediate effects on soil moisture and temperature compared to its effects on soil C concentration, C:N ratio, and pH. Second, functional diversity and identity in terms of litter N content were removed, given that we did not develop clear hypotheses directly linking this trait to soil moisture or temperature. Therefore, the model for soil moisture took the following form:

(2) $$\text{Soil}\;\text{moisture}\;∼\;\left(\begin{array}{c} \text{FDis}\;\text{Above}1+\text{FDis}\;\text{Above}2+\text{FDis}\;\text{Below}1+\text{FDis}\;\text{Below}2+\text{SR}+G\\ +\text{RB}+\text{RP}+\text{CWM}\;\text{Above}1+\text{CWM}\;\text{Above}2+\text{CWM}\;\text{Below}1+\text{CWM}\;\text{Below}2+\\ \text{Elevation}+\%\text{Sand} \end{array}\right)\times \text{Time}+\text{random}\left(\text{Block}\right)+\text{random}(\text{Treatment)}$$

We assumed that above‐ground mechanisms would play the determining role for soil temperature; therefore, below‐ground variables were not integrated into its analysis. However, the fraction of photosynthetically active radiation reaching the ground (fPAR) was added to its model, which took the following form:

(3) $$\text{Soil}\;\text{temperature}\;∼\;\left(\begin{array}{c} \text{FDis}\;\text{Above}1+\text{FDis}\;\text{Above}2+\text{SR}+G+\text{CWM}\;\text{Above}1+\text{CWM}\;\text{Above}2+\;\\ \text{fPAR}+\text{Elevation}\;+\%\text{Sand} \end{array}\right)\times \text{Time}+\text{random}\left(\text{Block}\right)+\text{random}\left(\text{Treatment}\right)$$

Multicollinearity arose in these linear models. G, RB, and RP (whether cumulated or not) are strongly correlated with *Time* (*r* > .85). We wanted to test the effects of all of these variables. Therefore, G, RB, and RP were scaled to an average value of zero for each year separately, making them independent of Time. Even so, in accordance with our first hypothesis, these variables still characterized differences in productivity among tree communities.

For each of the five soil variables and each one of their measurements within a mixture, a deviation from the expected value was calculated. These relative diversity effects can be detected when an observed value in a mixture is greater (synergistic effect) or smaller (antagonistic effect) than the expected value, based upon the respective monocultures. The relative land output (RLO) was used as the metric and was obtained as:

(4) $$\text{RLO}=\frac{\text{Observed}}{\text{Expected}}$$

where the observed value within a mixture is divided by the expected value. The latter is a weighted mean of the concomitant monoculture observations that was obtained with:

(5) $$\text{Expected}=\sum \left(m_{i}\times weight_{i}\right)$$

where the average value for each species *i* is obtained from its monoculture within the same block (*m_i_*) and is weighted by its relative abundance within the mixture (*weight_i_*). To characterize the relative abundance of each species, G cumulated over the years (Figure S3) was used for soil C concentration, C:N ratio and pH, while G was used for soil moisture and temperature. Student's *t*‐tests determined whether RLO differed from one, which is the null hypothesis.

Given that there was a great number of statistical tests in the present work (159 in total, i.e., each factor in each model, the correlation test and the *t* tests), a certain number of null hypotheses are bound to be rejected merely due to chance (~8 on average with α = .05). To avoid these Type I errors and misleading interpretations, a “false discovery rate” method was used, which is less conservative compared to “family‐wise error rate” methods such as the Bonferroni method (Benjamini & Hochberg, 1995; see section 3.1 in their paper for the procedure). Although a more conservative method is better for avoiding false discoveries, it can lead to a greater number of Type II errors, that is, “missed discoveries.” Prior to applying the “false discovery rate” method, 39 out of the 159 tests were significant (α = .05). Following the method application, 5 of these 39 significant results were deemed Type I errors and were not further considered as being significant.

Prior to all analyses, necessary transformations were applied to the data to obtain normal distributions. In the models, all independent variables were normalized (*µ* = 0, *σ^2^* = 1) to obtain standardized regression coefficients. All analyses were performed in *R* version 3.5.3 (R Core Team, 2018) with the package *lme4* (Bates et al., 2015) for general linear mixed‐effect models.

## 3 RESULTS

**FIGURE 3:**
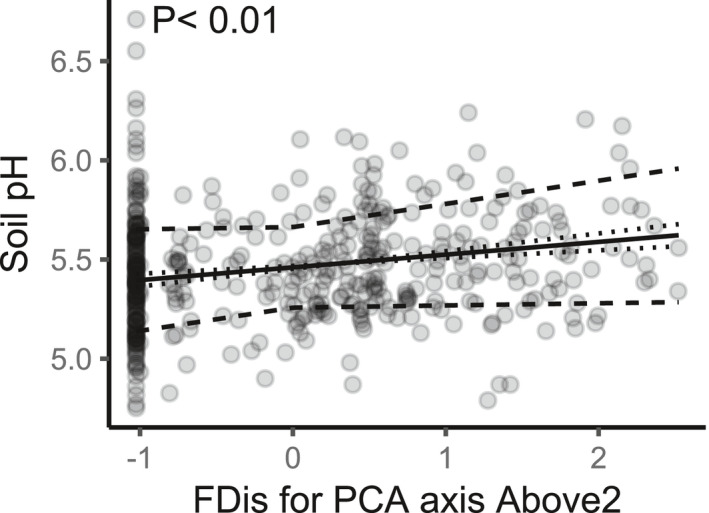
Soil pH as a function of functional dispersion (FDis) for the second axis of the above‐ground trait PCA (Above2), for all plots and the three sampling years (*N* = 427). FDis is normalized. Continuous lines are fitted values from the final general linear mixed‐effect models (Table 1). Dashed lines are the 95% confidence bands around the slope. Dotted lines represent the positive interaction with time (slope slightly steepens from 2012 to 2019)

**FIGURE 4:**
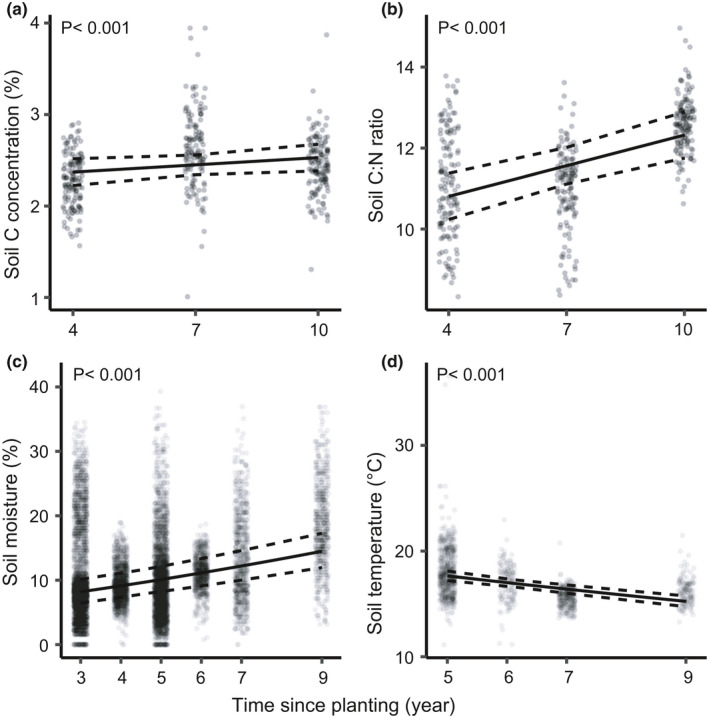
Effect of time since planting on soil C concentration (a), C:N ratio (b), moisture (c), and temperature (d). Continuous lines are fitted values from the final general linear mixed‐effect models (Table 1). Dashed lines are the 95% confidence bands around the slopes. Data points are jittered horizontally to improve visual interpretation

Tree diversity and identity indices did not affect soil C concentration (Table 1). Above‐ or below‐ground tree productivity did not affect soil C. There was a positive correlation between soil C and moisture (*r* = .24; *N* = 144; *p* < .01) and sandier soils had lower soil C concentration. Soil C:N ratio did not respond to tree variables (Table 1). Temperature had an inconsistent effect on the C:N ratio. Soil pH was affected by many diversity indices, but most of these effects were inconsistent through time, that is, negative at the beginning and positive at the end of the experiment, except for the consistently positive association between soil pH and functional dispersion (FDis) for the second axis of the above‐ground trait PCA (Figure 3). This diversity metric reflects the “fast‐slow” gradient (see Figure 2). Soil pH responded positively to plot elevation, that is, the variable controlling for micro‐topography. This could be the result of increased rhizodeposition in these plots given that biomass allocation to below‐ground parts is more important for trees in elevated plots (Martin‐Guay et al., 2020). Time positively affected soil C concentration and soil C:N ratio, and it was the strongest predictor in the models explaining C:N ratio (Table 1; Figure 4a,b).

**FIGURE 5:**
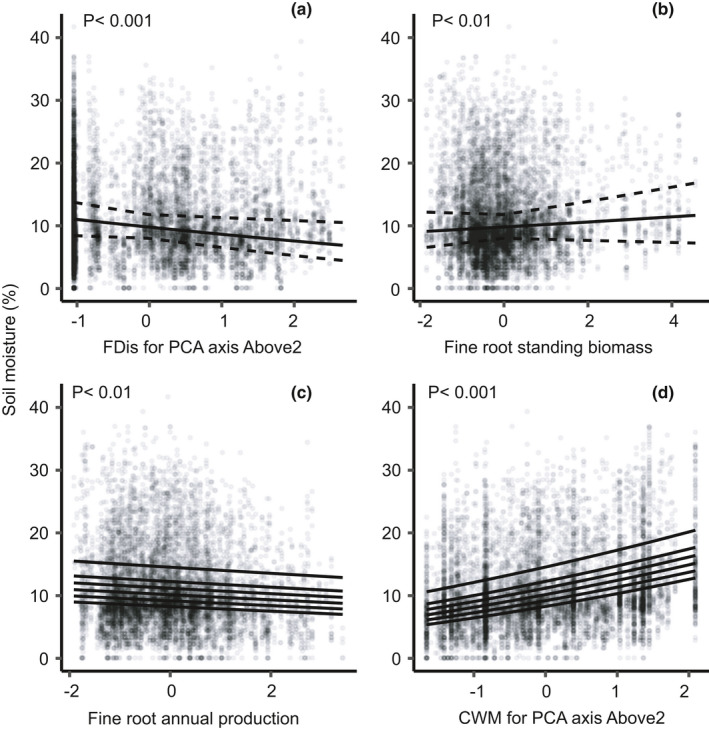
Soil moisture as a function of functional dispersion (FDis) for the second axis of the above‐ground trait PCA (Above2) (a), standing fine root biomass (b), fine root annual production (c), and community‐weighted mean (CWM) for Above2 (d), for all plots and the six sampling years (*N* = 9134). Independent variables are normalized. Continuous lines in A and B are fitted values from the final general linear mixed‐effect model (Table 1) and dashed lines are the 95% confidence bands about the slopes. For predictors with significant interactions (c and d), continuous lines show fitted values for each year separately. In each panel, the bottom line is the earliest sampling year, the second line the second earliest year, and so on (see Figure 4c)

Soil moisture, as a dependent variable, was predicted by several variables (Table 1), including fine root annual production, which was associated with drier soils, and standing fine root biomass, which was positively related to soil moisture (Figure 5b,c). Many functional identity and diversity metrics, as well as ground‐level basal area, were positive or negative predictors in early years and changed in later years (Table 1; predictors with a null effect overall, but significant interaction). These metrics included the community‐weighted mean (CWM) for the first axis of the above‐ground trait PCA (Above1), the CWMs for the first and second axes of the below‐ground traits PCA (Below1 and Below2), and functional dispersions (FDis) for Above1 (see Figure 2 for both PCAs). CWM for the second axis of the above‐ground trait PCA (Above2) had a consistent positive effect, which increased slightly through time (Figure 5d). Thus, soils were wetter under tree communities with more shade‐tolerant and slow‐growing species (i.e., large values on Above2, see Figure 2). Moreover, drier soils were associated with FDis for Above2, suggesting that greater tree diversity in terms of growth and shade‐tolerance strategies reduced soil moisture (Figure 5a). Soil moisture was also affected by soil texture and micro‐topography at the plot level, and these effects became more pronounced with time (Table 1). Time was the strongest predictor (*β* = .26), with soil moisture increasing through time (Figure 4c). Therefore, afforestation increased soil moisture, but less effectively in sandier soils and elevated plots.

**FIGURE 6:**
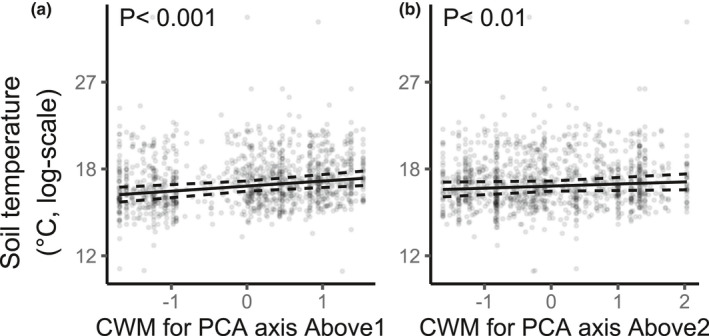
Soil temperature as a function of community‐weighted mean (CWM) for the first axis of the above‐ground trait PCA (Above1) (a) and CWM for the second axis (Above2) (b), for all plots and all sampling years (*N* = 1147). Independent variables are normalized. Continuous lines are fitted values from the final general linear mixed‐effect model (Table 1) and dashed lines are the 95% confidence bands about the slopes

Community‐weighted mean for Above1 and Above2 had positive effects on soil temperature (Figure 6a,b, respectively), indicating that temperature increased with greater relative abundances of broadleaf species (large values on Above 1) or of slow‐growing and shade‐tolerant species (large values on Above2). The long‐term trend was that soil temperature decreased with time (*β* = −.10, Table 1; Figure 4d).

The relative diversity effects that were calculated with relative land output (RLO; observed / expected) were absent for soil C concentration, C:N ratio, and temperature (respective *t*‐tests: *t* = 1.65, *df* = 282; *t* = −1.71, *df* = 764; *t* = 2.00, *df* = 282; both *p* > .05 and false discovery rate method). Thus, their observed values within mixtures were equivalent, on average, to their expected values that were obtained from the respective monocultures, that is, confirmation of the null hypothesis. Within mixtures for soil pH, observed values were 1.4% greater than expected values (*t* = 5.14, *df* = 284, *p* < .001). In contrast, observed soil moisture values were 1.2% smaller than expected (*t* = −12.5, *df* = 6085, *p* < .001).

## 4 DISCUSSION

### 4.1 Tree diversity effects on soils

Tree diversity metrics (species richness and functional diversity) did not explain variability in soil C concentrations when metrics were used as predictors of the soil C concentration, or when mixture values were compared to monoculture values. Therefore, we reject our first hypothesis. This was unexpected because the soil organic carbon (SOC) concentration in mixed forests is greater than monocultures, according to a global meta‐analysis that was based upon 74 studies with 261 independent observations (Chen et al., 2020). However, average stand age of the forests in the meta‐analysis was 31.1 y (SD: ±30.6 year); our evaluation was performed after 10 years. With 50‐cm spacing, our experiment was conceived for very rapid biotic interactions. Four years after establishment, strong crown interactions occurred (Williams et al., 2021); a single soil core in mixtures frequently had the roots from different species (unpublished data from Archambault et al., 2019); and litter decomposition, soil respiration, and microbial activity were already affected by diversity (Jewell et al., 2017; Khlifa et al., 2017). This would suggest that the diversity mechanisms affecting SOC were present, but their effects would need to cumulate over a longer period of time to become detectable. This is not surprising considering that a large proportion of the C in the topsoil can be very old, that is, at least 50 years old (Balesdent et al., 2018).

Some mixtures in the IDENT—Montreal site had greater above‐ground biomass than their corresponding monocultures (Tobner et al., 2016), but this does not affect soil C concentrations since there was no effect of tree communities with greater ground‐level basal area on the soil C concentration. Likewise, the observation of lower standing fine root biomasses in mixtures (Archambault et al., 2019) appears to be inconsequential for the soil C cycle, given that fine root biomass or production did not affect soil C. If the soil C concentration is directly proportional to the C input: C output ratio, this would suggest that this ratio remained constant across the different tree communities, meaning that increased C inputs for a community came with increased C outputs.

An earlier report of above‐ground productivity being positively correlated with microbial biomass at this site (Khlifa et al., 2017) suggests that greater above‐ground productivity was balanced by an increase in microbial respiration, indicating a compensatory response in the plant–soil system. This proposal is consistent with greater soil CO_2_ emissions (Jewell et al., 2017) and greater microbial biomass and respiration (Khlifa et al., 2017) in mixtures than monocultures, which is in line with other plant diversity experiments (Chen et al., 2019). In addition, microbial communities became more versatile in terms of carbon substrate use in mixtures, which could be explained by a diversification of below‐ground niches with tree diversity (Khlifa et al., 2017). Yet, there was no difference in the soil C:N ratio and temperature among tree species richness and functional diversity. Therefore, we assume that tree diversity has likely changed neither the N content of litter inputs that reach the mineral soil nor temperature‐dependent reactions, but tree diversity could alter other soil conditions that would favor increased microbial activity.

This assumption is supported by the fact that soil pH increased in tree communities that were more diverse in terms of growth strategies and shade tolerance. The model predicted an average soil pH of ~5.4 in monocultures and an average ~5.6 under the most diverse mixtures, based upon these traits. When comparing soil pH in mixtures to an expected value that is based upon the respective monocultures, pH was 1.4% (95% CI: [1.09, 1.19]) greater than expected. This positive effect of diversity on soil pH could mean increased microbe growth and eventually increased stable SOC from microbial necromass (Lange et al., 2015). Tree mixtures may be disproportionately increasing soil pH because they supply more litter and basic cations as a result of overyielding (Tobner et al., 2016), but tree biomass metrics did not have a direct impact on soil pH. Hence, this deviation from the expected value must be caused by chemical interactions in the mixed litter. The “fast‐slow” gradient was strongly correlated with leaf dry matter content (LDMC; see Figure 2), which means that tree communities with diverse LDMC had higher pH than communities with either low or high LDMC.

There was a decrease in soil moisture, which is inconsistent with increased microbial activity considering that precipitation–interception experiments generally reduce microbial biomass (Zhou et al., 2018) and that soil moisture was identified as the main driver globally of microbial biomass (Serna‐Chavez et al., 2013). Functionally diverse tree communities had slightly lower soil moisture, that is, diverse in terms of growth strategies and shade tolerance; furthermore, soil moisture in mixtures was 1.2% lower than expected than the respective monocultures. Tree moisture uptake can be greater in these functionally diverse communities due to niche partitioning (e.g., Mueller et al., 2013; Schwendenmann et al., 2015) or due to enhanced productivity. Nevertheless, vertical root segregation was deemed unlikely in a previous study, given that root depth was unaffected by species richness (Archambault et al., 2019). Therefore, these results offer mixed support for our second hypothesis. Indeed, we expected increased pH and moisture in mixtures, while moisture was lower than expected. Yet, the small size of these deviations from expected values, including that of soil moisture, could reveal that interspecific interactions are not very important for soils at this early stage of plantation development.

### 4.2 Tree identity effects on soils

Functional identity had no effect on soil C concentration, C:N ratio, or pH, which implies that the conifer percentage (taxonomic identity) did not affect these soil properties. In contrast, functional identity affected both soil moisture and temperature. Communities with fast‐growing species had lower soil moisture content. These communities could have taken up more moisture or relied more upon water in the uppermost horizon (moisture was measured with 12‐cm long TDR rods) than the slow‐growing communities (Schwendenmann et al., 2015). This is consistent with the negative effects of fine root production (measured in the topmost 15 cm) on soil moisture. Fast‐growing conifers (broadleaf litter was rapidly decomposed, based upon our on‐site observations) could have created litter mats, thereby limiting water infiltration (Facelli & Pickett, 1991).

Communities with slow‐growing and shade‐tolerant species exhibited higher soil temperatures. The same was true for tree communities with broadleaf‐deciduous species, meaning a negative effect of conifer relative abundance. These communities might have let more light through their canopies, although the amount of light reaching the ground did not seem to influence soil temperature. Litter accumulation under coniferous species can also create an insulation effect, reducing soil temperature (Facelli & Pickett, 1991). In another tree diversity experiment, the presence of pines increased soil surface temperature at night and, as a consequence, decomposition (Gottschall et al., 2019). Our temperatures were only recorded during the day; thus, the negative effects of conifers on soil temperature could be reversed during the night.

Given that conifer abundance only affected soil temperature and no other soil variable, support for our third hypothesis is equivocal. Based upon increased microbial activity under deciduous species (Khlifa et al., 2017), we expected that coniferous‐evergreen species would create soil conditions unfavorable to soil microorganisms, that is, increased C:N ratio, reduced pH, moisture, and temperature. Moreover, a negative effect on soil C was expected considering that afforestation using broadleaf species is usually more efficient than conifers at accumulating SOC in the mineral soil (Laganière et al., 2010). One caveat, however, is that the organic layer acts as the main C sink within the first three decades following planting due to recalcitrant litter, which often originates from coniferous species (Mayer et al., 2020; Vesterdal et al., 2013), and soil C was measured only in the uppermost mineral soil layer in the present study. When considering both organic and mineral layers together, the literature is still not clear on whether soils contain more carbon under coniferous or broadleaf species (Augusto et al., 2015; Mayer et al., 2020). Although coniferous species were less productive in our experiment, including the organic layer could have resulted in greater SOC overall under these species. In addition, Schmidt et al. (2011) found that subsoil C (>30 cm depth) could have an unexpected importance, since it represents half of all SOC; moreover, it was as sensitive to land‐use changes as was the topmost 30 cm of mineral soil. Nevertheless, forest floor C is often deemed to be more susceptible to disturbance and, thus a less interesting avenue for long‐term stabilization of SOC (Mayer et al., 2020; Vesterdal et al., 2013). The uppermost soil would be the first mineral layer to be affected by tree litterfall and the 0–10 cm depth often had the greatest fine root densities in the different tree communities of the experiment (Archambault et al., 2019).

### 4.3 Indirect tree effects on soil carbon through soil microclimate

In the current study, we assumed that the trees would have more immediate effects on soil microclimate (moisture and temperature) than on soil chemistry (C concentration, C:N ratio, and pH), which we deemed to be more gradual due to slow and incremental processes, for example, repeated annual litterfall (see Figure 1). This assumption was supported since both microclimate variables (soil moisture and temperature, *R^2^* = .24 and .27, respectively) responded to trees, while only one chemistry variable (soil pH, *R^2^* = .11) responded to trees. Therefore, it was possible to assess whether there were indirect effects of trees through soil moisture and temperature by testing the effects of soil moisture and temperature on soil C concentration, C:N ratio, and pH. Soil temperature did not affect any of these variables, even though a negative effect on soil C was expected based upon soil‐warming experiments (Sun et al., 2011).

Soil moisture was positively correlated with soil C concentration. It was tested using correlation since two causal relationships were deemed possible. On one hand, microbial biomass responds positively to soil moisture, based upon experiments altering precipitation (Zhou et al., 2018), and microbe‐growth efficiency subsequently could lead to increased stable SOC in the form of microbe‐derived SOM (Cotrufo et al., 2013). On the other hand, SOC and SOM are intrinsically linked, and SOM content can affect soil water‐holding capacity. The effect on soil moisture could be the most rapid means by which tree composition would affect the C cycle. For example, the abundance in slow‐growing and shade‐tolerant species could have had a positive effect on soil C concentration, through its positive effect on soil moisture. The plausibility of this causal structure was shown in a post hoc analysis using structural equation modeling (Appendix S2). Caution must be exercised when interpreting these indirect effects; while ~20% of the variability in soil moisture was explained by tree composition, only ~6% of the variability in SOC concentration was explained by soil moisture (Figure S4).

Although tree diversity and identity did not have a direct effect on soil C, the presence of trees was significant since there was a slightly positive effect of time on soil C concentration. Soil C sequestration is expected when afforestation is conducted on cropland (Laganière et al., 2010), as is the case in our experiment. One of the hypotheses that has been advanced to explain this phenomenon is the modification of microclimatic conditions by trees, which concurs with the reduced soil temperature and increased soil moisture with time (Figure 4). However, this positive effect on soil C might be an artifact considering that the first sampling of soil C was done using the top 15 cm of mineral soil while only the top 10 cm was used in the last two samplings. Indeed, soil C concentration sharply decreases along the soil profile. At least 10 years must elapse before an effect of afforestation on SOC can be detected according to Laganière et al. (2010), while five years are usually needed before SOC concentrations becomes greater in plant mixtures (including forest mixtures) compared to the respective monocultures (Chen et al., 2020). Our results indeed suggest that a decade may not be long enough to detect the effects of tree identity or diversity on the C cycle. There is poor support for our fifth hypothesis regarding strengthening effects of trees through time. Indeed, there were only two instances in which tree effects slightly strengthened, that is, the positive effect of diversity in terms of growth strategy and shade tolerance on soil pH (Figure 3) and the positive effect of slow‐growing and shade‐tolerant species on soil moisture (Figure 5d).

Since there was no direct effect of either tree identity or diversity on two of the five soil variables, notably soil C concentration, coming to any conclusion regarding the relative importance of tree identity and diversity is premature. The only support for our fourth hypothesis is that tree identity was more important for both soil moisture and temperature (based upon standardized coefficient sizes and the significance of predictors), while soil pH was the only variable for which tree diversity was more important with only 11% of its variability explained by the model predictors (Table 1). This is consistent with the general observation that was made by Mayer et al. (2020) in their literature review regarding the greater importance of tree identity compared to tree diversity.

## 5 CONCLUSION

While soil C concentration and C:N ratio did not respond directly to tree identity or diversity ten years after planting, soil pH, temperature, and moisture did respond. These effects could be the first effective way by which tree composition can alter different soil processes that are linked to the C cycle. Higher pH and lower moisture in diverse communities could create a shift in microbial communities together with microbial catabolism and anabolism, which will affect plant‐derived SOM decomposition and the accumulation of stable SOC (Lange et al., 2015). Feedback effects on tree growth could also occur due to increased cation‐exchange capacity or water stress. However, for these expected effects to be detected eventually, a decade‐old experiment might not be appropriate, as suggested by the absence of direct effects on soil C concentration. Finally, our results seem to point toward a greater importance of tree identity compared to diversity (Mayer et al., 2020). Soil microclimate was mostly affected by life‐strategy traits and the deciduous‐evergreen functional gradient. Therefore, forest managers might have to prioritize species with the desired traits before capitalizing on interspecific interactions.

## CONFLICT OF INTEREST

None declared.

## AUTHOR CONTRIBUTION

**Marc‐Olivier Martin‐Guay:** Conceptualization (lead); Formal analysis (lead); Investigation (lead); Methodology (lead); Writing – original draft (lead). **Michaël Belluau:** Data curation (supporting); Writing – review & editing (equal). **Benoît Côté:** Writing – review & editing (equal). **Ira Tanya Handa:** Data curation (equal); Writing – review & editing (equal). **Mark D. Jewell:** Writing – review & editing (equal). **Rim Khlifa:** Data curation (equal); Writing – review & editing (equal). **Alison D. Munson:** Data curation (equal); Writing – review & editing (equal). **Maxime Rivest:** Data curation (equal); Writing – review & editing (supporting). **Joann K. Whalen:** Writing – review & editing (equal). **David Rivest:** Conceptualization (lead); Funding acquisition (lead); Project administration (lead); Writing – review & editing (equal).

## Supporting information

Supplementary Material

## Data Availability

Data for the models (https://doi.org/10.5061/dryad.ksn02v75n) and the functional traits (https://doi.org/10.6084/m9.figshare.13118132.v1) are publicly available.
