# Supplementary material for: Tree identity and diversity directly affect soil moisture and temperature but not soil carbon ten years after planting
Source: Ecol Evol. 2022 Jan 12;12(1):e8509. doi: 10.1002/ece3.8509 (PMC8809433; doi:10.1002/ece3.8509)
Supplement: Supplementary file 1 — Supplementary Material [file ECE3-12-e8509-s001.docx]

# SUPPORTING INFORMATION: *Tree identity and diversity directly affect soil moisture and temperature but not soil carbon ten years after planting*

## Appendix S1. Accounting for prior climatic variability when evaluating soil moisture and temperature in field plots

Weather conditions were always different in the days before soil moisture and temperature were measured in field plots. Therefore, we used the daily average of air temperature and precipitation from the nearest weather station (Ste-Anne-de-Bellevue 1; 45°25'38" N, 73°55'45" W; Meteorological Service of Canada 2019) to account for recent climatic variability.

We used simple linear regressions to explain how soil moisture and temperature responded to prior air temperature and precipitation conditions. Soil moisture was plotted against the summed daily precipitation during the previous 5, 10, 20 or 30 d (including the measurement day). Similarly, soil temperature was plotted with the average air temperature during the same period (last 5, 10, 20 or 30 d). Coefficients of determination (*R^2^*) for the linear regression indicated the strength of the relationship between the soil variable and recent precipitation or fluctuations in air temperature. To avoid predictions with negative values for the percentage soil moisture, which has a zero-bounded distribution, soil moisture was ln-transformed prior to curve-fitting.

Total precipitation during the previous 10 d was the best predictor that explained 20.5 % of the variability in soil moisture (Fig. S1). Average air temperature in the previous 5 d described 58.1 % of the variability in soil temperature (Fig. S2). As expected, both relationships were positive. Before further analysis, we standardised the soil moisture and temperature as follows:

*Standardized value_i_ = Average measured value + Climate model residual_i_* (Eq. S1)

where the standardized value for measurement *i* is the average in-field measurement (*Average measured value*) plus the residual for this measurement *i* from the best-fit linear regression model (*Climate model residual_i_*). Since the linear regression model relied upon ln-transformed soil moisture, it was necessary to ln-transform the *Average measured value* of soil moisture and then back-transform the result, to get the *Standardized value_i_* in percent soil moisture. This procedure makes each measurement of soil moisture or temperature less dependent on recent precipitation events or temperature fluctuations.


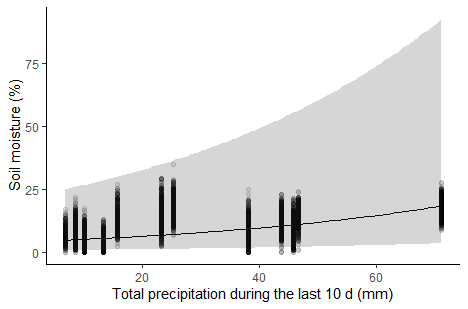


**Figure S1.** Soil moisture as a function of total precipitation during the previous 10 d (soil sampling day included). The line shows the best-fit linear regression (using ln-transformed values of soil moisture) and the shaded area represents the 95% prediction interval. *N*= 9134. Line equation: *ln(y) = 0.02x + 1.48*


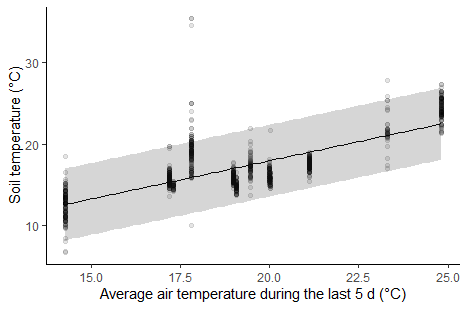


**Figure S2.** Soil temperature as a function of average air temperature during the previous 5 d (soil sampling day included). The line is the best-fit linear regression and the shaded area represents the 95% prediction interval. *N*= 1147. Line equation: *y = 0.94x - 0.82*

## References

Meteorological Service of Canada (2019) Past weather and climate - Historical data. URL: https://climate.weather.gc.ca/historical_data/search_historic_data_e.html. (downloaded on 25 May 2020)

**Figure S3.** Example of the calculation of ground-level basal area (the bold line) cumulated during the years preceding soil samplings. Data are from a plot with mixed *Pinus resinosa* and *Picea rubens*. The grey and white areas under the bold line represent the percentage of ground-level basal area occupied in autumn by *P. resinosa* and *P. rubens*, respectively. The ground-level basal area from 2009 until one of the years associated with soil sampling (vertical dashed lines) is a proxy for the tree cumulative influence on soil C, C;N ratio and pH. The relative influence of a particular tree species reflects the proportion of the ground-level basal area occupied by that species. Accordingly, the proportion of this area occupied by each species represents its relative influence on soil properties. With this method, the cumulative influence of each species can be more precisely characterised. This graph illustrates that tree species make unequal contributions to the ground-level basal area, since *P. resinosa* (grey area under the curve) was more abundant during the measurement period and likely produced more litterfall than *P. rubens* (white area under the curve). Since soil C, C:N ratio and pH were measured in the spring of 2019, we could relate the soil data to the ground-level basal area cumulated until autumn 2018.

## Appendix S2. Post hoc analysis reveals that soil C concentration was associated with soil moisture fluctuations related to tree composition

Tree composition may affect soil C concentration directly and indirectly. We evaluated the indirect effects using post hoc analysis with structural equation modelling to assess the association between trees, soil moisture and temperature, and the soil C concentration.

In the main paper, soil moisture was shown to be correlated significantly with soil C concentration, while soil temperature did not explain soil C (Table 1). Therefore, only soil moisture was tested in the structural equation model. In the general linear mixed model from the main paper, there were only two metrics of tree identity or diversity that predicted significantly soil moisture consistently through time, i.e., the functional dispersion (FDis) derived from the second axis of the above-ground trait PCA (see Fig. 1), and the community weighted mean (CWM) for the same axis (Table 1). Hence, this post hoc analysis using structural equation modelling only contained these two variables. Non-significant links were removed through a backward stepwise process, and the most parsimonious model is in Fig. S4.

**Figure S4.** Most parsimonious structural equation model showing how soil C concentration could be affected indirectly by tree identity through soil moisture (Model-fit test: *χ^2^*= 3.150, *df*= 1, *P*= 0.076). Significance levels of the standardized path coefficients (number next to the arrow) are: ** *P*< 0.01 and *** *P*< 0.001. Grey arrow was included in the *a priori* model, but is not considered to be influential because it was not significant (α= 0.05). The *R^2^*-value of soil moisture and C concentration indicates the proportion of variance explained by the model.
